# What Pushes University Professors to Burnout? A Systematic Review of Sociodemographic and Psychosocial Determinants

**DOI:** 10.3390/ijerph22081214

**Published:** 2025-08-01

**Authors:** Henry Cadena-Povea, Marco Hernández-Martínez, Gabriela Bastidas-Amador, Hugo Torres-Andrade

**Affiliations:** Facultad de Educación, Ciencia y Tecnología, Universidad Técnica del Norte, Ibarra 100105, Ecuador; mahernandezm@utn.edu.ec (M.H.-M.); agbastidas@utn.edu.ec (G.B.-A.); hptorres@utn.edu.ec (H.T.-A.)

**Keywords:** burnout syndrome, mental health, university professors, work performance, academic environment

## Abstract

Burnout syndrome is a growing concern in higher education, affecting the psychological well-being and performance of university professors. This systematic review presents a narrative synthesis of findings from quantitative studies on sociodemographic and psychosocial determinants of academic burnout. Following PRISMA 2020 guidelines, sixty peer-reviewed articles published between Jan 2019 and May 2024 were selected from Scopus and Web of Science. Inclusion criteria required validated psychometric instruments and exclusive focus on university faculty. Methodological quality was assessed using the Newcastle-Ottawa Scale and CASP checklist. Data from approximately 43,639 academic staff were analyzed. Key risk factors identified include excessive workload, lack of institutional support, and workplace conflict. In contrast, collegial support, participative leadership, and job satisfaction functioned as protective elements. Variables such as age, gender, academic rank, and employment stability significantly influenced burnout vulnerability. While general patterns were observed across studies, differences in design and sampling require caution in generalization. The evidence supports the implementation of integrated strategies encompassing mental health programs, workload regulation, participatory governance, and culturally responsive approaches. These findings inform the development of institutional policies aimed at preventing burnout and fostering academic well-being. Future research should adopt longitudinal and cross-cultural designs to further explore burnout trajectories and support educational reform.

## 1. Introduction

Burnout syndrome, also referred to as professional burnout, has emerged as a growing focus in scientific research due to its detrimental impact on mental health and job performance among professionals [1,2,3,4,5]. One of the earliest to conceptualize this condition was Freudenberger [6], who described it as a state of emotional exhaustion, depersonalization, and reduced personal accomplishment—particularly among individuals in helping professions [7,8,9,10]. Given its multifactorial origins, burnout must be understood through both sociodemographic and psychosocial lenses, which shape its development and progression [11,12,13,14,15].

In an academic context, burnout has attracted increasing concern due to the multifaceted nature of university professors’ responsibilities. Teaching, research, administrative tasks, and student advising place significant emotional and psychological demands on faculty members [13,16,17,18,19]. These challenges are compounded by institutional expectations related to publishing, securing external funding, and meeting performance metrics, all of which contribute substantially to burnout risk [16,18].

A considerable body of research has identified correlations between burnout and sociodemographic factors such as age, gender, marital status, and years of professional experience [7,15,20,21,22,23]. Younger or less experienced academics often face greater vulnerability, largely due to precarious employment conditions and limited coping resources [7,15,21,23]. Gender-based differences have also been noted, influencing both the manifestation of burnout symptoms and the strategies used to manage them in academic environments [14,20,24].

Despite the growing attention to burnout among university faculty, existing research remains fragmented—scattered across various disciplines, geographic regions, and methodological designs [16,25,26]. There is a pressing need for an integrative synthesis that consolidates this dispersed knowledge and clarifies the role of sociodemographic and psychosocial variables. Such an effort would not only enrich theoretical frameworks but also inform institutional policy and guide the development of targeted, context-sensitive interventions aimed at reducing burnout and fostering academic well-being [2,15,27].

Accordingly, the purpose of this review is to systematically examine the sociodemographic and psychosocial factors associated with the incidence and severity of burnout syndrome among university professors, offering a comprehensive overview of the current scientific evidence.

## 2. Materials and Methods

This study is a systematic literature review conducted in alignment with the PRISMA 2020 guidelines [28,29], ensuring methodological rigor, transparency, and reproducibility. The primary aim of this review was to examine the sociodemographic and psychosocial factors that influence the incidence and severity of burnout syndrome among university professors. The review process adhered to the standard PRISMA phases—identification, screening, eligibility, and inclusion—with clearly defined criteria applied consistently at each stage.

### 2.1. Eligibility Criteria

Inclusion and exclusion criteria were clearly established to guide the selection of relevant studies, as outlined in Table 1. Eligible studies met the following parameters: publication date between 2019 and 2024; publication type limited to peer-reviewed journal articles; target population consisting of university professors; and the use of validated instruments to assess burnout. Only empirical studies indexed in Scopus or Web of Science that examined sociodemographic or psychosocial determinants of burnout were included. Gray literature, preprints, non-peer-reviewed sources, and articles unrelated to the higher education context were excluded. These criteria were applied consistently throughout the review process to ensure the relevance, reliability, and scientific quality of the evidence analyzed.

### 2.2. Search Strategy

The literature search was conducted in two electronic databases: Scopus and Web of Science. Peer-reviewed studies published between 2019 and May 2024 were included. Specific search terms and combinations of keywords related to burnout syndrome, as well as sociodemographic and psychosocial factors in university professors, were used to formulate the canonical search equation: (“University Teachers” OR “Professor” OR “academic staff” OR “college faculty” OR “higher education instructors”) AND (“Psychosocial Factors” OR “demographic factors” OR “psycho” OR “psychological factors” OR “psychosocial influences” OR “soci*” OR “social factors” OR “sociodemographic variables” OR “socio-economic factors”) AND (“Burnout” OR “Job Burnout” OR “burnout*” OR “occupational burnout” OR “work-related burnout”). The following filters were applied in both databases: (1) articles published between 2019 and May 2024; (2) peer-reviewed scientific articles indexed in international databases; (3) studies using validated instruments to measure burnout syndrome; and (4) studies focused on university professors. The last search was conducted on 5 May 2024. No other sources such as reference lists, gray literature, or registries were used.

### 2.3. Study Selection Process

The study selection process was carried out in three stages: identification, screening, and inclusion. In the first stage, all relevant articles were retrieved from the selected databases. In the second stage, independent reviewers assessed the titles and abstracts of the retrieved articles to determine their initial eligibility. Articles that did not meet the inclusion criteria were excluded. In the third stage, the articles selected in the screening phase were fully evaluated to confirm their eligibility.

All records retrieved were screened independently by two reviewers at each phase of the selection process (title/abstract and full-text review). Discrepancies were resolved through discussion and consensus. Automation tools were used to assist with duplicate removal and preliminary filtering by publication year and article type, but final inclusion decisions were made manually.

**Figure 1:**
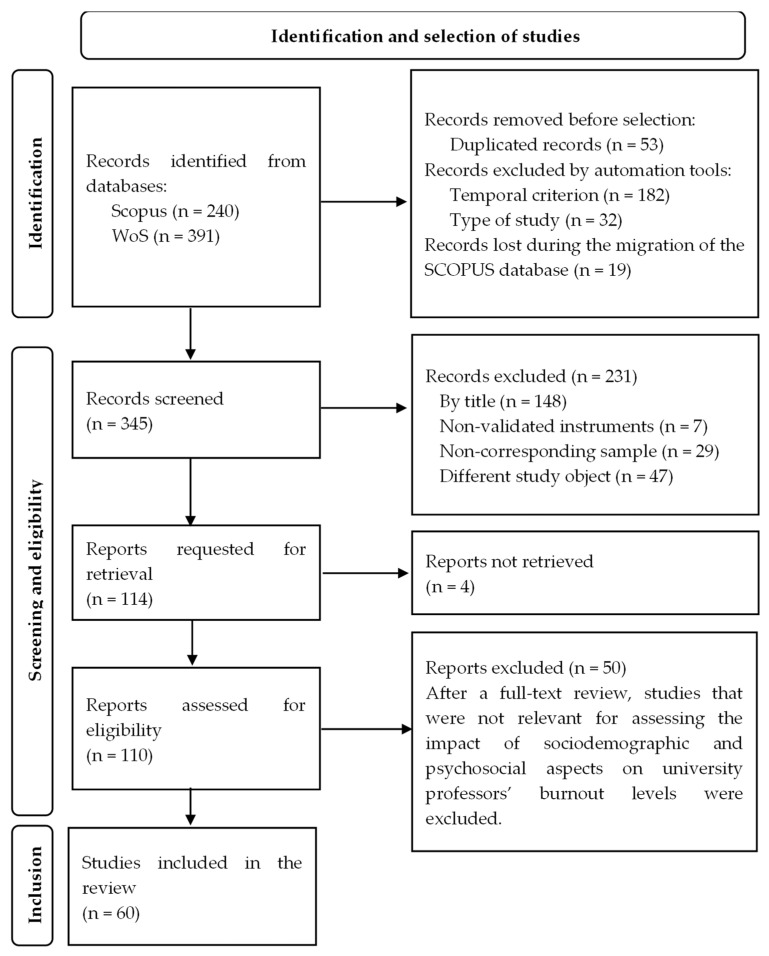
Flow diagram adapted from the PRISMA 2020 guidelines [29], outlining the multi-stage selection process, including identification, screening, eligibility, and inclusion phases, along with reasons for study exclusion.

Upon downloading the SCOPUS database, the platform indicated 138 records; however, after reviewing the file, 119 records were found. After full-text review, 50 studies were excluded from the final synthesis. Although these studies initially met the eligibility criteria, they did not reach the minimum quality threshold required by the assessment tools (Newcastle-Ottawa Scale or CASP) and were therefore discarded. Given that the reason for exclusion was consistent across cases, individual citations are not listed. The full selection process is shown in Figure 1.

### 2.4. Data Extraction

A standardized form was created for data extraction from the selected studies using the PARSIFAL digital tool. The extracted information includes bibliographic details (author, year of publication, country), study objective, study design, sample and demographic characteristics, sociodemographic and psychosocial factors assessed, measurement instruments used, findings related to burnout, conclusions, and recommendations.

Data extraction was carried out jointly by three reviewers. The process involved collaborative review and agreement on all extracted elements. To ensure accuracy, the extracted data were validated through repeated verification and cross-checking. When necessary, study authors were contacted to obtain missing information or clarify uncertainties in the reported data. The PARSIFAL tool was used to organize and document all extracted information in a systematic and traceable manner.

The primary outcomes for which data were sought included: (1) prevalence and levels of burnout syndrome, (2) associations between sociodemographic factors (e.g., age, gender, marital status, academic rank) and burnout, and (3) associations between psychosocial factors (e.g., job satisfaction, institutional support, workload, internal conflicts) and burnout. For each study, all reported results compatible with these outcome domains were extracted, regardless of the measurement instrument used or the time point of assessment. When multiple analyses or subgroups were presented, the most relevant and clearly reported results were prioritized, as determined by consensus among the reviewers.

In addition to outcome-related data, the following variables were extracted from each study: (1) bibliographic information (author, year, country), (2) study design and methodological quality, (3) sample characteristics (size, age distribution, gender composition, academic field), (4) measurement instruments used to assess burnout, (5) funding sources (when reported), and (6) journal indexing and language. When information was missing or unclear (e.g., participant characteristics or funding), assumptions were avoided and such cases were either documented as “not reported” or, when feasible, clarified through direct contact with the study authors.

### 2.5. Data Analysis

A categorical analysis was conducted to synthesize the findings of the included studies. Sociodemographic and psychosocial factors were grouped into thematic categories. Each category was analyzed to identify common patterns, differences, and relationships with the incidence and severity of burnout syndrome in university professors.

Studies were grouped into two thematic domains for synthesis: (1) sociodemographic factors associated with burnout, and (2) psychosocial factors associated with burnout. Eligibility for each synthesis was determined by the presence of at least one measurable variable aligned with the corresponding domain. The grouping process was conducted during data extraction, and studies reporting both types of factors were included in both syntheses. No studies were excluded from synthesis once they met the inclusion criteria outlined in Section 2.1.

No data transformation or conversion procedures were required for this review, as the synthesis was narrative and categorical in nature. In cases where essential summary statistics or specific data points were missing from the articles, the information was either marked as “not reported” or, when feasible, clarified through direct communication with the study authors. All data were synthesized as reported in the original studies.

To facilitate the synthesis and interpretation of findings, results from individual studies were tabulated using summary tables. A representative subset of six studies was presented in the main text (Table 2), selected based on methodological rigor, clarity of findings, and geographical diversity. The complete dataset, comprising all 60 included studies, was organized in Supplementary File S1. Tabulated data included authorship, year, country, sample characteristics, burnout instruments, key findings, and identified sociodemographic and psychosocial factors.

Although a quantitative synthesis was not performed, heterogeneity among studies was explored narratively by categorizing results according to sociodemographic and psychosocial domains. Differences in instruments used, regional context, academic rank, and institutional support were considered when interpreting patterns of association with burnout. This qualitative approach allowed for the identification of recurring themes and contextual variations in the included studies.

No sensitivity analyses were conducted, as the review employed a narrative and categorical synthesis without quantitative aggregation of data. As such, the robustness of the results was instead supported through cross-validation by multiple reviewers, thematic consistency across studies, and transparency in the presentation of inclusion criteria and data extraction procedures.

No formal tool (e.g., GRADE) was used to assess the certainty or confidence in the body of evidence due to the narrative and descriptive nature of the synthesis. However, general judgments about evidence strength were based on methodological quality (assessed via NOS and CASP), consistency of findings across studies, and the presence of potential biases. As a result, the overall certainty of the evidence was considered moderate to low.

To improve interpretability, the factors identified across the included studies were classified as either risk factors or protective factors, based on the direction and consistency of their reported associations with burnout. Risk factors referred to variables consistently linked to a higher prevalence or greater severity of burnout symptoms—such as excessive workload, lack of institutional support, or job insecurity. Conversely, protective factors were those associated with reduced burnout levels or buffering effects—such as high job satisfaction, collegial relationships, or a healthy work–life balance. This classification was established through consensus among the reviewers during the data extraction and synthesis process, taking into account the clarity of findings and theoretical coherence across studies.

### 2.6. Quality Assessment of the Included Studies

To ensure methodological rigor and minimize bias, the quality of each included study was independently evaluated using two well-established appraisal tools, selected according to the study design: the Newcastle-Ottawa Scale (NOS) for observational studies and the Critical Appraisal Skills Programme (CASP) checklist for qualitative studies. These tools enabled a structured and reproducible assessment of internal validity, clarity of research aims, sample adequacy, measurement precision, analytical rigor, and transparency in reporting.

Within the PARSIFAL platform (developed by the Universidad de los Andes, located in Bogotá, Colombia), each study was scored using standardized criteria adapted from the NOS and CASP frameworks. A three-tier scoring system was applied: 1 point for full compliance with a criterion, 0.5 points for partial compliance, and 0 points for non-compliance. Only studies that achieved at least 75% of the total possible score were included, ensuring that the final synthesis was based exclusively on robust and high-quality evidence.

All assessments were conducted independently by two reviewers. Any discrepancies were resolved through discussion and consensus. The entire quality assessment process was documented and archived within the PARSIFAL system for full traceability. Studies that did not meet the quality threshold were excluded and omitted from data extraction and synthesis. Since all retained studies met the minimum quality standards, individual quality ratings are not reported in detail.

### 2.7. Assessment of Reporting Bias

No formal assessment of reporting biases, such as publication bias or selective outcome reporting, was conducted due to the narrative and categorical nature of this systematic review, which did not include a quantitative meta-analysis. However, efforts were made to minimize potential reporting biases by implementing a comprehensive search strategy across two major databases (Scopus and Web of Science) and applying a clearly defined inclusion and exclusion criteria. Nevertheless, it is acknowledged that restricting the search to peer-reviewed published articles may introduce a potential risk of publication bias, as studies with non-significant or unfavorable results may remain unpublished.

### 2.8. Effect Measures

Due to methodological heterogeneity and variability among the included studies—particularly in study designs, sample characteristics, and measurement instruments—a quantitative synthesis or meta-analysis was not feasible. Consequently, standardized effect measures (e.g., odds ratios, risk ratios, or mean differences) reported in individual studies were neither extracted nor pooled. Instead, a narrative and categorical synthesis was conducted to systematically identify, classify, and interpret the sociodemographic and psychosocial factors associated with burnout syndrome among university professors. This approach facilitated a nuanced understanding of the complex, multifaceted nature of burnout while accounting for the methodological inconsistencies across studies.

### 2.9. Registration and Protocol

This systematic review was not registered in any public database or registry (e.g., PROSPERO). A formal protocol for this systematic review was developed using the PARSIFAL platform. The protocol was used for internal planning purposes and is not publicly accessible. No amendments were made to the original protocol developed in the PARSIFAL platform during this systematic review.

## 3. Results

Results are presented in the form of descriptive narratives and summary tables. Heterogeneity of the studies was discussed, and potential sources of bias were explored. The most relevant results of the systematic review are presented below, based on the study’s objectives.

### 3.1. Contextual Characteristics

Out of the 60 articles analyzed, a descriptive analysis was conducted regarding publication year, language, and country of origin. The highest number of studies were conducted in 2023 (28%), followed by 2019 (20%), 2020 (18%), 2021 (17%), and 2022 (12%). As of May 2024, 5% of the studies had been published.

In terms of language, most of the publications were written in English (87%), while others appeared in Spanish (8%), Portuguese (3%), French (1%), and Russian (1%).

Geographically, 20% of the studies were conducted in China, followed by Spain and Ecuador (10% each). Brazil, Colombia, Mexico, Peru, and Turkey each accounted for approximately 5% of the sample. The United States, Italy, and Portugal contributed about 3% of the studies. Finally, 1.7% of the studies were conducted in countries including Argentina, Belgium, Switzerland, Cameroon, Egypt, France, Kazakhstan, Lithuania, Nigeria, Pakistan, Poland, Sweden, Thailand, and Ukraine.

### 3.2. Methodological Characteristics

The studies reviewed primarily use quantitative methodologies, accounting for 93%, while the remaining 7% correspond to systematic reviews. An analysis of the burnout measurement instruments used in the reviewed studies shows that the Maslach Burnout Inventory (MBI) is the most widely used (85.00%). Other instruments include the Job Burnout Scale (1.67%), Cuestionario para la Evaluación del Síndrome de Quemarse por el Trabajo (CESQT) (3.33%), the China Occupational Burnout Scale (1.67%), the Oldenburg Burnout Inventory (OLBI) (3.33%), the Burnout Assessment Tool (BAT) (1.67%), the COPSOQ II Professional Burnout Scale (1.67%), and the Scale of Work Engagement and Burnout (SWEBO) (1.67%). These results highlight the preference for the MBI in burnout measurement, while other instruments, though less represented, remain significant.

### 3.3. Findings from Individual Studies

Table 2 presents a condensed synthesis of six representative studies included in this systematic review. These studies were selected based on methodological rigor, clarity of findings, geographical diversity, and relevance to the research objectives. They exemplify the diversity and methodological quality of studies that addressed multiple dimensions of burnout simultaneously, incorporating both sociodemographic and psychosocial aspects. The table highlights key information such as authorship, country, sample characteristics and instrument used, main findings, and the factors assessed. While this selection offers a succinct yet meaningful overview of prevailing trends in the literature, the full dataset encompassing all 60 reviewed studies—spanning a broader array of contexts, methodological approaches, and variables—is available as Supplementary Materials (Supplementary File S1). This strategy ensures clarity and coherence within the main text, while providing readers with comprehensive data for detailed examination and verification.

Detailed results from each study, including sample size, burnout prevalence, measurement instruments, and associations with sociodemographic and psychosocial factors, are presented in Supplementary File S1. Where available, summary statistics and effect estimates were included; however, many studies reported findings narratively or without confidence intervals.

In addition to the representative studies summarized in Table 2, a broader synthesis of the key findings across all 60 studies included in this systematic review is presented in Table 3. This consolidated summary highlights the most frequently reported sociodemographic and psychosocial factors associated with burnout among university professors.

The table categorizes each factor by type (sociodemographic or psychosocial), indicates the number of studies in which it was identified, and classifies it as either a risk or protective factor. Furthermore, a representative citation is provided for each factor, allowing readers to trace specific studies in which these associations were analyzed. This summary improves the readability and accessibility of the findings by offering a panoramic view of the recurring determinants of burnout in academic settings.

As shown in Table 3, burnout among university professors is consistently associated with both individual sociodemographic characteristics and psychosocial working conditions. This summary facilitates a clearer understanding of the most prevalent risk and protective factors reported across the literature.

### 3.4. Analysis of Sociodemographic and Psychosocial Factors

**Figure 2:**
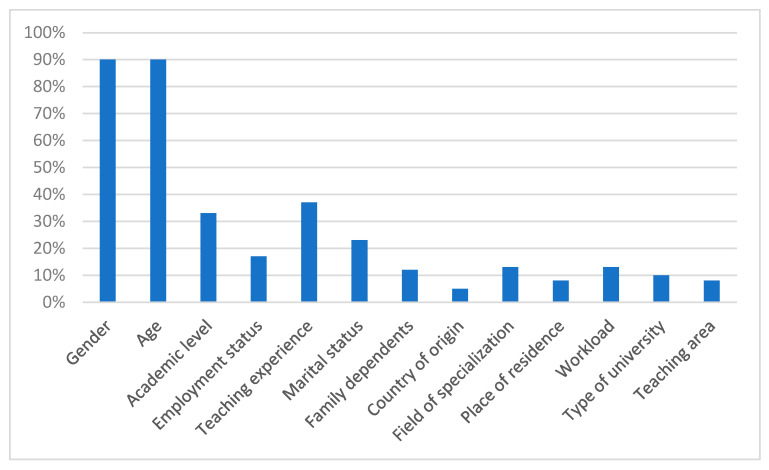
Sociodemographic factors.

This synthesis includes 60 studies that explored the relationship between sociodemographic (e.g., age, gender, marital status, academic rank) and psychosocial factors (e.g., job satisfaction, workload, organizational support, psychological well-being) and burnout syndrome among university professors. The studies varied in geographical scope, with representation from Latin America, Europe, Asia, and Africa, and sample sizes ranging from fewer than 100 to over 7565 participants. Most were cross-sectional in design and used validated instruments, such as the Maslach Burnout Inventory (MBI) or the Oldenburg Burnout Inventory (OLBI). All included studies met the minimum methodological quality threshold (≥75%) and were classified as low or moderate risk of bias, ensuring a solid basis for the synthesis. Figure 2 shows the percentage distribution of various sociodemographic factors associated with burnout syndrome in university professors.

Beyond the identification of recurrent sociodemographic and psychosocial determinants, the synthesis of the 60 studies reveals notable divergences and interactions that nuance the understanding of burnout among university professors. For instance, while gender and age consistently appear as dominant variables, the interpretation of their influence varies across cultural and institutional contexts. In several Latin American studies [37,38,39], female professors reported higher emotional exhaustion levels, primarily due to compounded domestic and professional responsibilities. However, in certain Asian contexts [40,41], younger male academics were also significantly affected, which some authors attributed to intense pressure to publish and limited career advancement opportunities.

Academic rank emerged as a contextual modifier of burnout vulnerability. In highly hierarchical systems [25,42], junior faculty reported greater depersonalization and lower personal accomplishment, often linked to unstable contracts and limited institutional agency. Conversely, in some European studies [16,43], senior professors experienced higher stress due to administrative overload and role ambiguity, suggesting that burnout risks may reappear at different career stages but for distinct reasons.

Interaction effects between sociodemographic and psychosocial dimensions were reported in several studies. For example, study 36 found that marital status moderated the impact of institutional support, with single professors being more susceptible to burnout when lacking collegial relationships. Likewise, study 27 highlighted that the detrimental effects of workload were exacerbated among professors without access to professional development programs, regardless of their gender or age.

Geographical comparisons also revealed regional disparities in the intensity and configuration of burnout predictors. In South American countries [7,9,44], burnout was often associated with systemic underfunding, high teaching loads, and limited access to mental health services. In contrast, North American and Western European studies [22,45] frequently emphasized psychosocial stressors such as research-teaching conflicts, perceived unfairness, and academic isolation. These findings suggest that, while burnout may manifest globally, its underlying causes are deeply embedded in local academic and policy environments.

Psychosocial variables were also found to co-occur and amplify each other. Study 17 demonstrated that low job satisfaction combined with poor work–life balance predicted a steeper decline in mental health, particularly in women. Meanwhile, study 29 underscored that internal conflicts—especially among departments with competitive research cultures—acted as a trigger for emotional exhaustion even in the presence of structural support mechanisms.

Although less frequent, some studies [24,26] drew attention to protective effects stemming from job autonomy and professional identity. Professors who reported a strong alignment with their academic mission, or who had greater decision-making power in their departments, showed notably lower levels of burnout—even in resource-constrained settings. These findings highlight the potential of non-material buffers against stress and suggest new avenues for policy design beyond financial investment.

These results not only confirm the relevance of common burnout predictors but also reveals complex interdependencies between individual, organizational, and contextual variables. The heterogeneity observed across studies underscores the importance of tailored interventions that consider local academic structures, career trajectories, and psychosocial dynamics rather than relying on one-size-fits-all solutions.

Several studies emphasize that burnout does not arise from isolated factors but rather from the interaction between sociodemographic and psychosocial dimensions. For instance, study 2 revealed that female faculty members under the age of 41 with family responsibilities reported significantly higher levels of emotional exhaustion, particularly when combined with high teaching loads and limited institutional support. Similarly, study 10 demonstrated that job dissatisfaction intensified burnout symptoms mainly among professors in lower academic ranks and fixed-term positions, suggesting that employment instability exacerbates the psychological burden associated with unrecognized professional contributions.

In study 46, the combination of high perceived stress and limited autonomy predicted elevated burnout levels more strongly than either factor alone. These findings indicate the need to interpret determinants of burnout not as linear predictors but as part of a multidimensional configuration in which personal vulnerabilities, role expectations, and institutional structures converge. Consequently, future intervention strategies should consider these synergies to more effectively target high-risk groups within academic settings.

While there is broad consensus regarding the core predictors of burnout, the review also reveals notable inconsistencies across studies. For example, study 42 found no significant gender differences in burnout levels, contradicting the patterns observed in many Latin American and European studies. This divergence was attributed to institutional policies in the sampled university that supported flexible scheduling and caregiving leave regardless of gender, suggesting that contextual factors may override typical demographic trends.

These discrepancies underscore the complexity of gender-related findings in burnout research. While many studies report that female university professors are more susceptible to emotional exhaustion—possibly due to increased emotional demands, difficulties balancing work and personal life, or role strain [10,24,32,38,46]—male professors are more frequently associated with elevated levels of depersonalization or diminished personal accomplishment. These patterns may reflect differences in coping mechanisms, societal expectations, or institutional dynamics [23,27,47]. The inconsistencies observed across studies likely arise from variations in measurement tools, cultural and regional settings, or sample demographics [10,24,48]. As such, gender should not be treated as a uniform risk factor; rather, its influence on burnout appears multifaceted and context-dependent, highlighting the need for further investigation [8,37,38].

Despite these overall patterns, contradictory findings emerged across several studies. For example, while many investigations reported higher levels of emotional exhaustion among female professors, others found no significant gender differences—or even higher burnout levels among male faculty in certain regions. Similarly, although early-career academics were frequently identified as a high-risk group, some studies indicated greater burnout among senior faculty, often attributed to heavier administrative responsibilities and declining institutional support. These discrepancies may stem from variations in study design (e.g., sample size, measurement instruments), institutional frameworks (e.g., tenure systems versus contract-based employment), or cultural norms surrounding gender roles, workload expectations, and available support structures. Such divergences underscore the need to interpret burnout as a context-sensitive phenomenon shaped by local academic environments and methodological diversity.

Moreover, while most studies reported workload as a primary risk factor, studies 13 and 20 found that academic burnout was more strongly associated with perceived injustice and lack of participation in institutional decisions, even in contexts where workloads were moderate. In study 34, burnout prevalence was unexpectedly low despite high job demands, which the authors linked to strong peer mentoring programs and a culture of collegiality. These inconsistencies underscore the importance of analyzing mediating and moderating variables, as well as local organizational dynamics, to avoid overgeneralization and to better contextualize burnout outcomes.

Gender and age factors (90% each) emerge as the most prominent variables, suggesting a strong correlation between these demographic aspects and burnout syndrome among university professors. This implies that gender and age are key determinants in the manifestation of this syndrome, possibly due to differences in gender roles and expectations, as well as the accumulation of stress with age [8,10,37,49]. Additionally, the academic level is significantly represented (40%), indicating that the level of academic training of professors influences their susceptibility to burnout. Higher academic levels may be associated with greater responsibilities and professional expectations [12,22,36,40].

Factors such as marital status (50%) and family responsibilities (30%) reflect the impact of personal and family obligations on the development of this syndrome. Professors with family duties and diverse marital statuses may face additional stress outside the workplace, which contributes to the onset of burnout [18,34,50]. Both job stability (20%) and workload (20%) are crucial factors. Educators facing precarious employment conditions or heavy workloads are more susceptible to experiencing burnout [8,22]. Teaching experience (30%) is also a relevant factor. Educators with more years of experience may encounter varying levels of burnout due to prolonged exposure to stress-inducing factors [11,40].

Other factors, such as country of origin (10%), field of training (5%), place of residence (10%), type of university (10%), and teaching area (5%), have a lesser representation, indicating that, while they do exert some influence, their impact is less pronounced compared to other factors. However, these variables may still contribute to burnout when combined with more dominant factors [19,37,41,51].

The analysis of Figure 2 highlights that gender and age are the most influential sociodemographic factors associated with burnout syndrome in university professors, followed by academic level and marital status. These findings emphasize the importance of considering both phenotypic characteristics and personal and professional responsibilities when addressing burnout in this professional group. Additionally, factors such as employment relationship, teaching experience, and family responsibilities also play a significant role and should be considered in interventions and strategies for preventing the syndrome.

**Figure 3:**
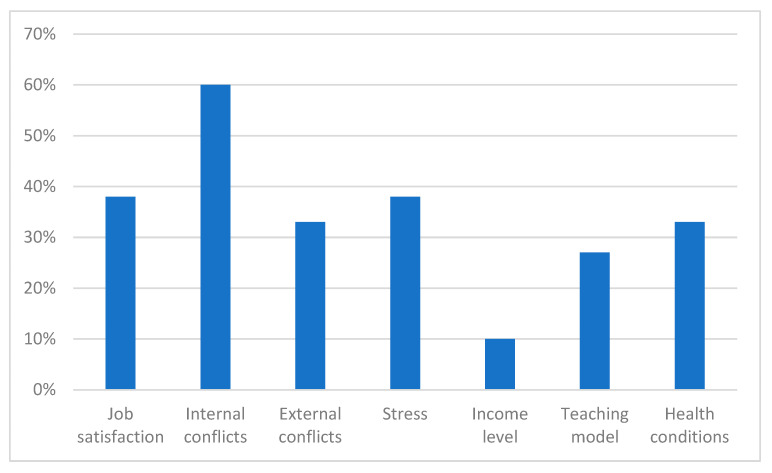
Psychosocial factors.

Figure 3 shows the percentage distribution of various factors associated with burnout syndrome in university professors.

Internal conflicts (60%) are the most significant factor, indicating that conflicts within the work environment, such as interpersonal issues or tensions with colleagues, are the most influential psychosocial factors associated with burnout syndrome in university professors [1,12,22,52,53,54]. Job satisfaction (38%) and work-related stress (38%) are equally represented, suggesting that both satisfaction with one’s work and stress resulting from job responsibilities are crucial in contributing to the development of this syndrome. Low job satisfaction can increase the risk of burnout, while chronic work-related stress is a well-known precursor to burnout [30,47,49].

Also, the factors of external conflicts (33%) and health conditions (33%) have significant representation, indicating that both conflicts outside the university environment (such as family or social issues) and physical or mental health conditions play an important role in the development of burnout [9,35,55]. The factor of teaching model (27%) refers to the pedagogical methodologies and techniques employed, which can influence the level of stress and exhaustion experienced by educators. An inadequate teaching model, or one that does not align with the needs of the professors, can significantly contribute to burnout [21,39].

Income (10%) is the factor with the least representation, suggesting that, although economic factors are relevant, their influence is lesser compared to the psychosocial factors mentioned earlier. While income can contribute to financial stress, it is not the predominant factor in the association with burnout [18,42,56].

The analysis of psychosocial factors shows that internal conflicts within the work environment are the most influential factors in the association with burnout syndrome in university professors. Job satisfaction and work-related stress are equally important, highlighting the need to improve the work environment and manage stress to prevent burnout. External conflicts and health conditions also have a significant impact, while the teaching model and income have a lesser but still relevant influence. These findings emphasize the need for a multifaceted intervention that addresses both the internal factors of the work environment and the personal and health circumstances of educators to mitigate burnout.

Most of the articles considered multiple factors, reflecting the complexity of the burnout phenomenon and the need for a multifaceted approach to understand its causes and effects on university professors. In response to this, the factors were categorized into sociodemographic and psychosocial. This detailed and categorized analysis provides a better understanding of how various sociodemographic and psychosocial factors influence the manifestation of burnout syndrome in this professional group, offering a foundation for more specific and contextually relevant interventions.

The analysis of the reviewed articles reveals a significant prevalence of burnout syndrome in university professors. It was observed that, in some studies, prevalence reached alarming levels, with a percentage showing high levels of emotional exhaustion, depersonalization, and low personal accomplishment [1,12,35,50]. This finding indicates a significant prevalence of burnout, especially among those professors with high job demands and low levels of institutional support [4,25,44,57].

Similarly, several studies have identified sociodemographic factors that influence the manifestation of burnout syndrome. Specifically, it has been found that age and teaching experience are critical variables, with younger professors and those with less experience reporting higher levels of burnout [20,36,39,58]. These sociodemographic factors appear to interact with psychosocial variables, such as social support and job satisfaction, to either exacerbate or mitigate the effects of burnout [7,10,26,41].

In terms of psychosocial factors, social support, both from colleagues and family, has been identified as a significant mitigator of burnout syndrome. Studies show that professors who report higher levels of social support tend to experience lower levels of emotional exhaustion and depersonalization [9,31,53,59]. Additionally, the perception of organizational justice and a positive work climate are crucial for reducing burnout symptoms in university professors [33,40,48,49].

Workload and lack of resources are frequently cited as factors contributing to the development of burnout. University professors facing excessive workloads, with administrative tasks added to their teaching responsibilities, show a greater tendency to experience burnout [2,11,37,52]. This phenomenon is exacerbated by the lack of adequate resources to perform their duties effectively, which increases pressure and work-related stress [3,8,13,34].

The analysis of the literature also highlights the influence of gender factors on the prevalence of burnout syndrome among university professors. Several studies have found that female professors tend to report higher levels of emotional exhaustion than their male counterparts, possibly due to the combination of professional and personal responsibilities [16,21,38,47]. This pattern suggests a need for gender-specific approaches in interventions designed to reduce burnout [23,24,42,45].

The relationship between leadership style and burnout has been explored in several studies, with findings suggesting that transformational and supportive leadership styles are linked to lower levels of burnout among university professors [18,27,35,57]. In contrast, authoritarian or laissez-faire leadership styles may elevate stress and emotional exhaustion, contributing to the higher prevalence of burnout [12,34,37,46].

A relevant psychosocial factor is job satisfaction. Studies have shown that higher job satisfaction is correlated with lower levels of burnout. Job satisfaction is influenced by various factors, including the perception of recognition and reward, the work–life balance, and opportunities for professional development [17,25,41,60]. When these elements are present, educators tend to experience fewer burnout symptoms [20,22,26,43].

Autonomy at work also plays a crucial role in mitigating burnout. Educators who have greater control over their tasks and work schedules exhibit lower levels of emotional exhaustion and depersonalization [2,19,36,39]. This finding highlights the importance of institutional policies that promote autonomy and flexibility in the academic work environment [9,13,49,59].

Training and professional development are identified as protective factors against burnout. Educators who engage in continuous training and professional development programs report lower levels of the syndrome, as these activities provide new strategies and tools for managing stress and enhancing classroom effectiveness [10,37,44,50]. These programs not only improve teaching competencies but also have the potential to increase job satisfaction and professional motivation [51,52,58,61].

Several intervention strategies have also been identified to reduce burnout syndrome among university educators. One of the most effective strategies is the implementation of wellness and mental health programs within educational institutions. These programs, which range from mindfulness sessions to psychological counseling, have significantly reduced emotional exhaustion levels and improved the overall well-being of educators [3,17,40,48]. The accessibility and regularity of these programs are crucial to their success [4,45,53,62].

Fostering an institutional culture of support and collaboration also emerges as a key intervention. Institutions that promote a collaborative work environment and provide strong institutional support, through inclusive policies and professional support networks, tend to have educators with lower levels of burnout [19,31,33,35]. The perception of support and recognition from the administration is essential for the emotional well-being of educators [25,26,37,49].

Another effective intervention is training in time management and coping skills. Studies indicate that when educators receive training in time management techniques and stress coping strategies, they experience a significant reduction in burnout levels [8,11,21,46]. These skills enable them to manage work demands more effectively and maintain a healthy balance between work and personal life [12,24,50,60].

Workplace flexibility and workload adjustments are key interventions. Allowing educators more flexibility in their schedules and adapting their workloads to prevent overload have been shown to reduce burnout levels [7,16,20,52]. Policies that support a balance between professional and personal life are particularly beneficial for educators with additional family responsibilities [2,9,13,63].

Support for research and continuous professional development is essential for mitigating burnout. Offering educators opportunities to engage in research projects and access ongoing training and professional development can enhance their job satisfaction and reduce the impact of burnout [22,39,47,57]. These opportunities not only enrich their professional growth but also increase their sense of accomplishment and purpose in their work [23,41,42,44].

These results highlight the importance of a multifaceted approach to addressing burnout syndrome in university professors, considering both sociodemographic and psychosocial factors. They also suggest intervention strategies implemented at the institutional level to improve professors’ well-being.

No formal sensitivity analyses were conducted, as the narrative synthesis was based exclusively on studies that met the predefined methodological quality threshold. The robustness of the synthesized results was supported by the consistency of patterns across studies from diverse geographical regions and academic contexts, all of which were assessed as low or moderate risk of bias.

Although no formal assessment tool (such as GRADE) was applied to evaluate the certainty of the evidence, the overall confidence in the synthesized findings was considered moderate. This judgment was based on the inclusion of studies with acceptable methodological quality (≥75%), the use of validated instruments, and the consistency of observed patterns across diverse geographical and academic contexts. However, the absence of standardized effect measures and the narrative nature of the synthesis limit the ability to rate the certainty as high.

### 3.5. Exploration of Variability

A narrative exploration of heterogeneity was conducted to identify patterns across studies based on geographical location, type of institution, and measurement instrument. For example, studies conducted in Latin America tended to report higher prevalence rates of burnout compared to those from Europe or Asia. In addition, the use of different measurement tools (e.g., MBI vs. OLBI) resulted in variability in reported outcomes and burnout dimensions. Institutional characteristics such as public vs. private status and faculty academic rank also contributed to differences in findings. These sources of heterogeneity were considered when interpreting the overall trends of the synthesis.

In addition to these factors, the variability in burnout outcomes was influenced by methodological design and sampling strategies. Cross-sectional studies with large representative samples [9,44,57] often reported more stable associations between sociodemographic variables and burnout levels, whereas studies with smaller or convenience samples [19,20,39] showed greater fluctuations in findings, especially regarding gender and academic rank. This heterogeneity may reflect differences in the reliability of the instruments or contextual influences not accounted for in more limited samples.

Language and cultural context also emerged as relevant moderators. For example, studies conducted in multilingual or bicultural settings [11,17,56] reported ambiguous interpretations of psychosocial constructs such as “organizational support” or “emotional exhaustion”, which may carry different connotations across institutional cultures. Furthermore, differences in how burnout dimensions were operationalized—such as varying cut-off scores for high vs. moderate burnout in MBI applications—contributed to discrepancies in reported prevalence, even when similar instruments were used.

Another source of heterogeneity stemmed from institutional mission and workload distribution. In research-intensive universities, burnout was more closely tied to publication pressure and grant competition [35,49], while in teaching-focused institutions, workload overload and classroom management were more prominent stressors [43,59]. Additionally, faculty working in medical or health sciences departments [37,40,52] tended to report higher depersonalization scores, possibly due to emotionally demanding interactions and dual teaching-clinical responsibilities.

Lastly, national policies and educational reforms were identified as contextual determinants of variability. For instance, in countries undergoing significant higher education restructuring (e.g., Ecuador, Turkey, and Ukraine), burnout levels spiked during periods of institutional transition [4,14,58]. Conversely, studies conducted in stable policy environments (e.g., Finland, Portugal) displayed lower emotional exhaustion, suggesting a buffering role of systemic consistency.

These findings underscore that heterogeneity in burnout outcomes is not merely an artifact of measurement but reflects real, context-dependent phenomena. Recognizing these layers of variability is essential to designing nuanced, context-sensitive strategies for addressing academic burnout in diverse higher education systems.

## 4. Discussion

The present study contributes meaningfully to the limited body of systematic reviews examining the influence of sociodemographic and psychosocial factors on burnout among university professors. By synthesizing evidence from diverse geographical and methodological contexts, this review strengthens the conceptual understanding of burnout as a multidimensional construct within academic settings. Crucially, it situates burnout not merely as an individual response to occupational strain but as the outcome of systemic, institutional, and sociocultural dynamics [11,31,39]. This perspective invites a reconceptualization of burnout beyond clinical symptomatology, framing it instead as an indicator of structural imbalance within the academic labor system [39,41].

Contemporary research on occupational burnout is increasingly grounded in theoretical models that explain how work conditions interact with psychological and physiological stress responses. A prominent framework applied across multiple studies in this review is the Demand–Control–Support (DCS) model developed by Karasek and Theorell. According to this model, burnout arises when employees face high psychological demands in combination with low decision latitude (control) and limited social support. These three dimensions were repeatedly reflected in the reviewed literature: high academic workload, minimal influence over institutional decisions, and insufficient collegial or managerial support emerged as key stressors among university professors [11,40,41,50,53]. The DCS model provides a useful lens for understanding how structural and interpersonal elements converge to influence burnout levels. Moreover, its explanatory power is enhanced when extended to educational settings, where task complexity and emotional labor intersect with organizational rigidity [31,39,53]. Several studies in this review implicitly align with the DCS framework by highlighting the protective role of autonomy and support in buffering the negative effects of workload and emotional exhaustion [41,44,50,58].

In addition to the Demand–Control–Support (DCS) model, the Job Demands–Resources (JD–R) framework offers a valuable theoretical lens for understanding the dynamics of burnout among university professors. This model conceptualizes burnout as the result of an imbalance between job demands—such as time pressure, emotional workload, and role conflict—and available job resources, including autonomy, social support, and access to professional development [11,40,50]. The JD–R framework expands on the DCS model by emphasizing the dual pathways of strain and motivation, suggesting that adequate resources not only mitigate burnout but also enhance engagement and resilience [41,46]. Several studies in this review implicitly align with the JD–R model by illustrating how lack of resources exacerbates emotional exhaustion, while strong institutional support functions as a buffer [7,25,31]. Incorporating this model helps explain the variability of burnout experiences across different academic settings and underscores the importance of resource-focused interventions.

Taken together, the Demand–Control–Support (DCS) and Job Demands–Resources (JD–R) models provide complementary perspectives on the factors driving burnout among university faculty. The DCS model highlights the crucial role of autonomy and social support in alleviating stress under high-demand conditions, while the JD–R model expands this view by emphasizing how available resources not only reduce strain but also promote engagement and resilience. The empirical findings in this review—such as the buffering effect of collegial support, the challenges posed by role ambiguity, and the influence of institutional culture—resonate strongly with the mechanisms outlined by both frameworks. By integrating these models, we gain a richer, more nuanced understanding of burnout as the result of a complex interplay between organizational constraints and resource availability, shaped further by personal and contextual factors.

Theoretical frameworks that inform burnout research underscore the role of academic precarity in shaping the psychological well-being of university faculty. This concept, which encompasses unstable employment conditions, limited contractual guarantees, and performance-driven evaluation systems, has been associated with heightened levels of stress and emotional exhaustion among professors [31,39,41]. Precarious employment arrangements—such as short-term contracts, limited access to research funding, and unclear promotion pathways—intensify feelings of job insecurity, erode professional identity, and diminish autonomy [44,50,53]. These structural stressors contribute to the erosion of faculty well-being and reinforce systemic inequalities within academic institutions, particularly in regions with limited institutional support or resource constraints [31,39,40]. Therefore, addressing academic precarity should be a strategic priority in institutional interventions aimed at reducing burnout risk.

One of the most consistent findings is the strong association between burnout syndrome and excessive workload. Multiple studies indicate that high work demands—exacerbated by insufficient resources and weak institutional support—significantly increase burnout levels among university professors [1,39,44]. The burden is not limited to the sheer volume of tasks but also includes their complexity, mode of delivery, and the level of responsibility involved, all of which adversely affect professors’ mental and physical well-being [1,11,62].

Psychosocial factors, particularly social support and the quality of interpersonal relationships within the academic workplace, also emerged as critical determinants. The reviewed studies consistently highlight that support from both peers and institutional leadership acts as a buffer against burnout [7,40,50,62]. A collaborative and supportive organizational climate not only mitigates perceived stress but also fosters job satisfaction and enhances professional engagement among faculty members [25,30,50].

Sociodemographic variables—such as age, gender, and years of professional experience—further shape vulnerability to burnout. Several studies report that younger and less experienced faculty members are especially susceptible, often due to limited coping strategies and the instability characteristic of early academic careers [2,41,58]. Conversely, other research suggests that senior academics may also experience elevated levels of burnout due to accumulated occupational stress and age-related health challenges [35,53]. Overall, workload, social support, and sociodemographic characteristics stand out as key determinants of burnout syndrome, reinforcing the need for targeted interventions that address these interrelated dimensions to enhance faculty well-being.

Further subgroup analyses within the reviewed studies highlight interaction effects between sociodemographic and psychosocial variables in shaping burnout risk. Several studies found that gender moderates the burnout experience, with women more often reporting emotional exhaustion, while men tend to exhibit higher levels of depersonalization [18,34,50]. Regarding age, younger faculty members showed greater vulnerability to workload-related stress, likely due to limited institutional experience, whereas older faculty faced increased risks linked to chronic stress accumulation and biological aging [19,34]. Additionally, professional status and institutional type influenced burnout severity: adjunct and non-tenured faculty at resource-constrained institutions reported higher levels of emotional exhaustion and job insecurity compared to their tenured peers [24,33]. These findings underscore the importance of intersectional analyses to capture how structural and psychological factors intersect across diverse academic subgroups.

These findings highlight the importance of examining burnout through the lens of intersecting factors rather than isolated variables. For instance, the impact of gender on emotional exhaustion can become more pronounced when combined with precarious employment or low social support, especially among early-career faculty [20,24,27,47,56]. Likewise, age-related vulnerabilities may be either mitigated or intensified depending on psychosocial conditions like collegial climate or autonomy [24,27,37,62]. In this way, sociodemographic factors often serve as moderators or amplifiers of psychosocial risks rather than acting as standalone predictors [24,27,47]. This perspective calls for a more integrated analytical approach that acknowledges the dynamic and cumulative nature of burnout vulnerability across different academic subpopulations.

At the individual level, promoting the development of coping skills and self-care strategies among university professors is critical. Empirical evidence suggests that training in stress management techniques—such as meditation and mindfulness—can effectively reduce burnout symptoms [8,37,57]. Equally important is encouraging a healthy work–life balance; programs that support physical activity and leisure have been shown to improve mental health outcomes among academic staff [20,26,48].

At the institutional level, cultivating a healthy and supportive work environment is essential. Universities are encouraged to implement comprehensive policies that reduce excessive workloads and enhance professional support systems. Recommended strategies include decreasing student–faculty ratios, improving access to pedagogical resources, and formally recognizing teaching contributions [3,36,59]. Additionally, offering continuous professional development opportunities is vital to maintaining academic motivation and fostering long-term institutional commitment [12,33,47].

Institutional characteristics—such as governance models, resource allocation policies, and administrative burden—exert a significant influence on burnout levels among university professors. Several studies emphasized how rigid hierarchical structures, excessive bureaucratic tasks, and inadequate pedagogical support contribute to emotional exhaustion and reduced professional satisfaction [39,40,41]. For example, universities with centralized decision-making processes and low faculty participation in governance often report lower levels of academic engagement and higher levels of occupational stress [25,41,44]. Conversely, institutions that implement shared governance models and inclusive leadership practices tend to foster greater psychological safety, thereby mitigating burnout risks [11,35,50].

Moreover, funding policies and institutional priorities also affect faculty workload and morale. Studies conducted in Latin American and Southern European contexts revealed that austerity-driven policies and performance-based funding mechanisms exacerbate faculty strain by incentivizing publication pressure and minimizing support for teaching innovation [31,39,40]. These institutional dynamics shape the working conditions in academia and must be central in the design of burnout-prevention strategies.

Another important institutional strategy involves the implementation of mentoring programs and access to psychological support services. The availability of trained counselors and peer support networks has demonstrated considerable efficacy in assisting professors experiencing elevated stress levels [34,38,52]. Furthermore, involving faculty members in decision-making processes and workload planning enhances their sense of agency and contributes to the prevention of burnout [4,9,21].

Cultural and regional variations must be carefully considered in the design and implementation of burnout interventions. Empirical evidence indicates that strategies are most effective when they are tailored to the cultural specificities of each academic context [10,17,49]. This calls for a flexible, context-sensitive approach that accounts for phenotypic and sociocultural differences shaping the experience and expression of burnout [24,27,49]. Accordingly, both individual- and institutional-level strategies are essential to mitigating burnout among university professors. Key actions include the adoption of evidence-based stress management programs, the promotion of supportive organizational climates, and the integration of culturally responsive practices to enhance faculty well-being.

At the individual level, strengthening coping strategies among university professors is a key priority. Several studies show that professors who actively engage in practices like mindfulness, physical exercise, or effective time management tend to experience lower emotional exhaustion and greater job satisfaction [11,21,26,33,55,63]. Training programs focused on evidence-based techniques—such as cognitive restructuring, emotional regulation, and resilience building—have proven effective in reducing stress and preventing burnout [11,21,63]. For example, some research [26,33] found that incorporating physical activity into daily routines significantly boosts mental well-being, while others [63] emphasize mindfulness as a powerful tool for managing stress responses. Therefore, higher education institutions should prioritize promoting self-care awareness through structured workshops and integrate stress management into faculty development curricula.

At the institutional level, the literature consistently highlights the need for systemic interventions focused on organizational culture, work structures, and policy reforms. Multiple high-quality studies identify excessive workload, lack of recognition, and limited decision-making autonomy as key contributors to burnout among university faculty [10,13,44,53,60,62]. Recommended approaches include adjusting teaching loads and easing administrative duties, especially for early-career and contingent staff [24,47,54]; adopting shared governance models to boost faculty involvement in institutional decisions [13,36,53,62]; establishing clear promotion paths and recognition systems that value teaching and mentoring efforts, which are often overlooked [14,40,49]; and ensuring access to professional development opportunities, including mentorship programs, to strengthen academic identity and reduce role ambiguity [2,5,42,45,54].

Another key area for institutional action is ensuring access to psychological support services. Research [5,27,42] has shown that confidential counseling, peer-support groups, and dedicated wellness centers are associated with lower levels of depersonalization and emotional exhaustion. Universities should integrate these resources into their human resources policies and promote mental health support by running destigmatization campaigns to normalize seeking help.

Importantly, adopting intersectional approaches in policy design is essential. For example, gender-responsive measures—such as parental leave and anti-harassment policies—as well as initiatives addressing structural disadvantages faced by ethnic minorities and contingent faculty, have been shown to help reduce burnout disparities among different groups [13,18,24,56]. Studies from the Global South and resource-constrained settings [10,17,60] highlight that austerity measures and performance-based funding can worsen burnout levels. Therefore, it is crucial for national and regional education policies to promote sustainable funding models, protect tenure-track positions, and establish clear, transparent evaluation criteria to safeguard faculty well-being.

Moreover, cross-regional differences in institutional governance, academic traditions, and resource availability play a significant role in shaping how burnout is experienced and managed. For example, studies from Latin America and Southern Europe often highlight higher stress levels linked to austerity measures, bureaucratic overload, and rigid hierarchies that restrict faculty autonomy. On the other hand, research from Nordic and Anglo-Saxon countries tends to focus more on the protective effects of work–life balance and participatory governance. These contrasts underscore the importance of understanding burnout not as a one-size-fits-all issue, but as a culturally rooted phenomenon influenced by national higher education systems, funding mechanisms, and societal attitudes toward academic work and mental health.

In addition to the institutional conditions already discussed, several structural barriers exacerbate burnout risk, particularly among underrepresented or marginalized faculty groups. Gender-based disparities, such as unequal pay, disproportionate caregiving burdens, and limited access to academic leadership roles, contribute to chronic stress and emotional exhaustion [39,41,58]. Furthermore, the persistence of institutional biases—including subtle forms of discrimination, lack of inclusive policies, and stigma surrounding mental health—undermines psychological safety and reduces the effectiveness of preventive strategies [7,53,57]. These dynamics are particularly harmful to women, early-career faculty, and those from ethnic or minority backgrounds, who often encounter cumulative disadvantage within academic systems [35,50,58]. Recognizing and addressing these systemic inequities is essential for developing burnout interventions that are both effective and equitable.

The practical implications derived from the reviewed studies underscore the importance of a holistic and multidimensional framework for addressing burnout (Table 4). Wellness programs that integrate both psychological and physical components appear more effective than isolated interventions [16,19,61]. Crucially, the success and sustainability of these initiatives depend on strong institutional support and active administrative involvement [13,19,45].

Although this review identified consistent patterns regarding the sociodemographic and psychosocial determinants of burnout syndrome among university professors, the overall certainty of the evidence remains moderate to low. This assessment is primarily due to the methodological heterogeneity of the included studies, variations in sample characteristics, and inconsistencies in the measurement instruments employed. Additional limitations include the absence of a formal assessment of publication bias and the scarcity of longitudinal data, both of which constrain the generalizability of the findings. While the synthesis provides valuable insights, its application across diverse educational contexts should be approached with caution.

The heterogeneity observed across the reviewed studies arises from several interconnected factors. First, sampling strategies varied greatly, ranging from nationally representative surveys to small, localized case studies with limited generalizability. Second, the use of different measurement tools—although the Maslach Burnout Inventory (MBI) was the most common, some studies relied on alternative or adapted scales—led to inconsistencies in how burnout was defined and the thresholds used to identify it. Third, contextual differences such as institution type (public versus private), resource availability, governance structures, and geographic location played a significant role in shaping the results. For instance, professors working in underfunded institutions in the Global South often face additional stressors not typically found in better-resourced settings, which tends to increase emotional exhaustion and job dissatisfaction. Finally, variations in theoretical frameworks—whether applying the Demand–Control–Support (DCS) model, the Job Demands–Resources (JD-R) model, or the Conservation of Resources (COR) theory—affected how burnout predictors were interpreted and prioritized. Together, these factors emphasize the need to approach synthesis with caution and highlight the importance of context-sensitive interpretations when drawing conclusions from aggregated data.

Moreover, several methodological limitations should be acknowledged. First, although the search strategy was carefully planned and carried out using two major academic databases (Scopus and Web of Science), limiting the search to these platforms might have excluded relevant studies found in other databases like ERIC, PsycINFO, or regional repositories. Second, by restricting the review to articles published only in English and Spanish, there is a risk of language bias, which may reduce the cultural and geographical diversity of the evidence. These limitations could have led to underrepresentation of perspectives from certain academic contexts or non-Western research traditions. Third, the review protocol was not prospectively registered in a public database such as PROSPERO, limiting external transparency. Despite adherence to a predefined protocol and rigorous methodological standards, prospective registration would have further strengthened the study’s credibility and minimized potential sources of bias.

It is recommended to create educational policies that prioritize the well-being, mental, and emotional health of professors as an institutional priority. This includes the allocation of adequate resources for psychological support programs, the flexibilization of workloads, and the promotion of a balance between work and personal life [18,46,60]. Institutions should also establish continuous evaluation mechanisms to monitor the impact of these policies and make necessary adjustments based on the results obtained [23,32,43].

Regarding recommendations for future research, it is crucial to expand the understanding of burnout by considering the diversity of educational, institutional, and cultural contexts. Comparative studies examining public and private institutions, centralized versus decentralized governance, and universities from high-, middle-, and low-income countries could elucidate how structural inequalities and organizational cultures modulate burnout risk. Likewise, investigations should adopt a multilevel perspective that includes macro-level policies, meso-level institutional dynamics, and micro-level psychological experiences to capture the complexity of burnout among university professors [14,41,63].

A longitudinal approach is especially needed to analyze the temporal progression of burnout symptoms, identify long-term predictors, and evaluate the sustained effects of preventive interventions. Mixed-methods designs would be particularly valuable in integrating quantitative data with in-depth qualitative narratives that reveal contextualized patterns of stress and coping mechanisms [55,56,63].

Another emerging area of inquiry concerns the impact of digital technologies on burnout experiences in academia. The rapid expansion of remote teaching, digital administration, and AI-based tools following the COVID-19 pandemic has created new psychosocial demands for professors. While some digital solutions (e.g., e-mental health apps, online wellness platforms) show promise in reducing burnout symptoms, others may contribute to screen fatigue, digital surveillance, and work–life boundary blurring [5,15,30,54]. Future research should evaluate the double-edged nature of digitalization—its risks and opportunities—with a focus on how hybrid and remote academic models influence faculty well-being.

Conceptually, future inquiries would benefit from engaging underutilized theoretical frameworks such as the Conservation of Resources (COR) theory, which emphasizes the depletion of personal and professional assets under chronic stress, or the Effort–Reward Imbalance (ERI) model, which focuses on the consequences of perceived inequity between academic effort and institutional recognition [2,41,50]. These models may help capture dimensions of burnout not fully addressed by the DCS or JD–R frameworks.

Moreover, special attention should be paid to underrepresented or structurally vulnerable academic populations, including adjunct and contingent faculty, early-career researchers, and professors from historically marginalized ethnic, gender, or socioeconomic backgrounds. Intersectional research designs are needed to reveal how overlapping systems of disadvantage contribute to differentiated experiences of burnout [13,38,56].

In summary, this review illustrates the multidimensional nature of burnout among university professors and reinforces the need for comprehensive, multilevel strategies to address it. The practical implications and proposed research directions offer a roadmap for future efforts to enhance the quality of academic life and safeguard faculty well-being across diverse educational systems. Urgent and coordinated action is needed to develop structural, cultural, and psychological interventions that can reverse the escalating erosion of academic mental health. By deepening our understanding and expanding the scope of future research, the academic community can foster more sustainable and equitable working environments for its members.

## 5. Conclusions

This systematic review presents a comprehensive and analytically robust synthesis of the sociodemographic and psychosocial determinants associated with burnout syndrome among university professors. Drawing on evidence from sixty peer-reviewed studies, the findings reveal burnout as a complex, multifactorial phenomenon shaped by the interplay of personal, professional, and organizational variables.

Key sociodemographic determinants—such as age, gender, academic rank, and employment stability—consistently influence susceptibility to burnout. Early-career academics, particularly those in precarious positions, exhibit elevated levels of emotional exhaustion and depersonalization, often linked to insufficient coping mechanisms and job insecurity. Nonetheless, senior faculty are not exempt, as cumulative occupational stress and increasing institutional demands may also contribute to burnout. Gender-based disparities are likewise prominent, with multiple studies reporting higher emotional exhaustion among female faculty, likely due to the dual burden of professional and domestic responsibilities.

Psychosocial factors further exacerbate vulnerability to burnout. High perceived stress, interpersonal conflict, and low job satisfaction are frequently cited as aggravating conditions—particularly in academic environments where excessive workloads are not counterbalanced by organizational support, autonomy, or recognition. Conversely, the presence of strong collegial networks, participative leadership, and institutional fairness appears to buffer the negative effects of stress, underscoring their protective role.

Most studies operationalize burnout through the Maslach Burnout Inventory (MBI), which facilitates diagnostic consistency but may also limit the incorporation of alternative conceptual frameworks. Moreover, while the reviewed studies reflect cultural and regional diversity, methodological heterogeneity—particularly in sampling strategies and measurement tools—warrants caution in generalizing findings across contexts.

Despite these limitations, this review makes a substantive contribution to the literature on academic burnout. It elucidates the multifaceted nature of the syndrome and demonstrates the interconnection between structural, psychosocial, and demographic dimensions within academic institutions. By integrating findings across diverse settings, the review affirms the global relevance of burnout and its systemic character within higher education.

The implications of this synthesis extend beyond academic inquiry. By mapping the institutional mechanisms that contribute to faculty distress, this review provides an evidence base to inform the development of more contextually responsive and structurally grounded policy interventions. It frames burnout not merely as an individual ailment, but as a manifestation of deeper organizational and cultural dynamics within academic labor.

These findings call for a paradigmatic shift in how academic well-being is addressed—from reactive, individualized responses toward anticipatory and systemic solutions. This review lays the groundwork for future longitudinal, interdisciplinary, and cross-cultural research aimed at further unpacking the complexity of burnout and guiding institutional transformations that promote faculty well-being and sustainability.

Effective burnout prevention requires interventions at both the individual and institutional levels. Individual-focused programs that incorporate mindfulness-based stress reduction, resilience training, and time management skills have demonstrated efficacy in reducing emotional exhaustion and enhancing faculty self-efficacy. At the institutional level, providing confidential counseling services, flexible workload policies, and peer support networks can mitigate the impact of chronic stress. Furthermore, leadership development aimed at fostering empathetic management and increasing faculty recognition is essential. Systemic strategies, including longitudinal monitoring of burnout indicators and participatory organizational reforms—such as revising evaluation criteria to appropriately value teaching, mentoring, and service—address structural stressors contributing to burnout. These multilevel approaches should be tailored to the specific context of each institution and continuously evaluated to ensure sustained effectiveness.

Future research should prioritize longitudinal and mixed-methods approaches to better capture the dynamic and multifaceted nature of burnout. There is a critical need to focus on underrepresented groups, including faculty in low-income countries, adjunct professors, and members of ethnic or linguistic minorities, whose experiences may differ substantially from those in more well-studied academic contexts. Comparative cross-national studies could provide valuable insights by disentangling the cultural, structural, and economic factors that shape burnout. Additionally, intervention-based research evaluating the effectiveness of specific policies, support programs, or organizational reforms would offer practical guidance for mitigating burnout. The adoption of standardized measurement tools across studies would further improve comparability and support the creation of global monitoring frameworks.

This review offers several actionable insights to inform institutional policies and interventions aimed at preventing academic burnout. First, early-career faculty—especially those on precarious contracts—should be prioritized for targeted support given their increased vulnerability to emotional exhaustion. Second, organizational reforms that foster participatory governance, strengthen collegial support, and promote work–life balance are critical to reducing psychosocial stressors. Third, investing in training on coping strategies and ongoing professional development has proven protective effects and should be a key focus for institutions. Lastly, implementing gender-sensitive measures and culturally responsive mental health programs is essential to address structural inequities and enhance faculty well-being across diverse academic environments.

## Figures and Tables

**Table 1:** Eligibility criteria for selecting studies.

	Inclusion Criteria	Exclusion Criteria
Type of Article	IC1: Scientific articles on the research field of burnout syndrome. IC2: Scientific articles published in indexed journals. IC3: Studies that used validated tools for measuring burnout syndrome in the studied population.	EC1: Studies that did not employ quantitative, qualitative, or mixed-method research. EC2: Articles that had not undergone peer review. EC3: Studies that did not use validated tools for measuring burnout syndrome in the studied population.
Time Frame	IC4: Studies published between 2019 and 2024.	EC4: Studies published before 2019.
Sample	IC5: University Professors.	EC5: Studies that did not investigate university professors.
Language	IC6: Ability to translate the language in which the article was originally written.	EC6: Lack of ability to translate the language in which the article was originally written.
Research Object	IC7: The relationship between sociodemographic or psychosocial factors and burnout syndrome.	EC7: Studies that did not align with the research objective.

Note. The inclusion and exclusion criteria were established prior to the screening process and were applied systematically and consistently throughout the review to ensure methodological rigor and relevance of the selected studies.

**Table 2:** Summary of six representative studies examining burnout in university professors.

Author, Year (Country)	Sample/Instrument	Main Findings	Sociodemographic Factors	Psychosocial Factors
[17] (China)	7565 professors/MBI (adapted)	Work stress predicted burnout; effect moderated by self-efficacy.	Age, gender, academic position	Workload, work–life balance, self-efficacy
[24] (Mexico; Colombia; Ecuador)	598 professors/MBI (Spanish version)	Spanish professors showed more burnout than Latin Americans.	Country of origin, gender	Perceived stress, organizational culture
[30] (United States)	6000 professors/MBI (EE and DP)	Academic affiliation lowered burnout; junior ranks had more burnout.	Academic rank, gender	Work demands, institutional support
[31] (Nigeria)	597 professors/MBI	High job demands linked to burnout; support mitigated effects.	Age, gender, academic rank	Job demands, autonomy, support
[32] (Belgium; Switzerland)	915 staff (professors)/BAT (short)	Job insecurity raised burnout; participation in decision-making reduced it.	Job type, employment status	Job insecurity, participatory culture
[33] (Pakistan)	1008 professors/OLBI	Psychological capital reduced burnout from workaholism.	Age, gender, employment status	Workaholism, psychological capital

Note. The studies included were selected based on methodological rigor, clarity of findings, and geographical diversity, and are presented as representative examples of the broader dataset.

**Table 3:** Consolidated summary of key factors associated with burnout among university professors across the 60 reviewed studies.

Factor	Type	No. of Studies	Risk/Protective	Example Citation
Marital status (single)	Sociodemographic	21	Risk	[1]
Gender (female)	Sociodemographic	39	Risk	[7]
Age (under 40)	Sociodemographic	33	Risk	[16]
Academic rank (lower positions)	Sociodemographic	19	Risk	[34]
Teaching experience (low)	Sociodemographic	15	Risk	[35]
Teaching–research conflict	Psychosocial	22	Risk	[12]
Perceived stress	Psychosocial	16	Risk	[18]
Work overload	Psychosocial	43	Risk	[36]
Coping style (avoidant)	Psychosocial	10	Risk	[37]
Self-efficacy	Psychosocial	12	Protective	[7]
Professional identity	Psychosocial	9	Protective	[25]
Supervisor support	Psychosocial	18	Protective	[26]
Psychological capital	Psychosocial	21	Protective	[33]
Work–life balance	Psychosocial	17	Protective	[38]
Job satisfaction	Psychosocial	24	Protective	[25]

**Table 4:** Summary of risk and protective factors linked to burnout among university professors.

Category	Risk Factors	Protective Factors
Sociodemographic	- Young or early-career academics - Precarious contracts - Gender-based inequalities	- Seniority with coping experience - Gender-sensitive policies
Occupational	- Excessive workload - Job insecurity - Lack of autonomy- Role conflict	- Flexible workload policies - Autonomy in academic tasks - Participatory governance
Psychosocial	- Low social support - High perceived stress - Interpersonal conflicts	- Collegial support - Strong professional identity - Access to mental health services
Institutional	- Centralized governance - Performance pressure - Inadequate recognition	- Inclusive leadership - Recognition of teaching and mentoring - Transparent evaluation models
Structural/Cultural	- Academic precarity - Discrimination and bias - Resource constraints in low-income settings	- Culturally responsive programs - Equitable policies - Diversity and inclusion frameworks

## Data Availability

The original contributions presented in this study are included in the article/Supplementary Materials. Further inquiries can be directed to the corresponding author.

## Supplementary Materials

The following supporting information can be downloaded at: https://www.mdpi.com/article/10.3390/ijerph22081214/s1, File S1: Summary of the main characteristics and findings of the studies included in the systematic review.

## Abbreviations

The following abbreviations are used in this manuscript:

BAT	Short version of the Burnout Assessment Tool
OLBI	Oldenburg Burnout Inventory
MBI	Maslach Burnout Inventory
EE	Emotional Exhaustion
DP	Depersonalisation
